# Release of sICAM-1 in Oocytes and *In Vitro* Fertilized Human Embryos

**DOI:** 10.1371/journal.pone.0003970

**Published:** 2008-12-18

**Authors:** Monica Borgatti, Roberta Rizzo, Maria Beatrice Dal Canto, Daniela Fumagalli, Mario Mignini Renzini, Rubens Fadini, Marina Stignani, Olavio Roberto Baricordi, Roberto Gambari

**Affiliations:** 1 BioPharmaNet, Department of Biochemistry and Molecular Biology, University of Ferrara, Ferrara, Italy; 2 Department of Experimental and Diagnostic Medicine, Laboratory of Immunogenetics, University of Ferrara, Ferrara, Italy; 3 BIOGENESI Reproductive Medicine Centre, Istituti Clinici Zucchi, Monza, Italy; 4 Biotechnology Center, University of Ferrara, Ferrara, Italy; University of Kansas Medical Center, United States of America

## Abstract

**Background:**

During the last years, several studies have reported the significant relationship between the production of soluble HLA-G molecules (sHLA-G) by 48–72 hours early embryos and an increased implantation rate in IVF protocols. As consequence, the detection of HLA-G modulation was suggested as a marker to identify the best embryos to be transferred. On the opposite, no suitable markers are available for the oocyte selection.

**Methodology/Principal Findings:**

The major finding of the present paper is that the release of ICAM-1 might be predictive of oocyte maturation. The results obtained are confirmed using three independent methodologies, such as ELISA, Bio-Plex assay and Western blotting. The sICAM-1 release is very high in immature oocytes, decrease in mature oocytes and become even lower in in vitro fertilized embryos. No significant differences were observed in the levels of sICAM-1 release between immature oocytes with different morphological characteristics. On the contrary, when the mature oocytes were subdivided accordingly to morphological criteria, the mean sICAM-I levels in grade 1 oocytes were significantly decreased when compared to grade 2 and 3 oocytes.

**Conclusions/Significance:**

The reduction of the number of fertilized oocytes and transferred embryos represents the main target of assisted reproductive medicine. We propose sICAM-1 as a biochemical marker for oocyte maturation and grading, with a possible interesting rebound in assisted reproduction techniques.

## Introduction

Successful embryo formation and implantation are critical steps during in vitro fertilization procedure. Unfortunately, approximately 10% of retrieved oocytes and fewer than 20% of transferred embryos result in a successful delivery [1]. Analysis of the embryo morphology in still one of the most common approaches of selection in assisted reproduction, with the obvious drawback of being to some extent subjective.

Accordingly, there is urgent need of biochemical markers facilitating the prediction of successful oocyte fertilization and implantation of the *in vitro* fertilized (IVF) human embryos. In this respect, the only biochemical marker so far proposed for the selection of the most promising embryo obtained by IVF is represented by the release of in vitro cultured embryo (24-, 48- and 72-hours embryo) of soluble HLA-G (Histocompatibility Leukocyte Antigen-G) molecules. This has been consistently reported by several groups [2]–[7]. Using Enzyme-Linked Immunosorbent Assay (ELISA) and Bio-plex approaches, these groups reported that high expression of soluble HLA-G is associated with higher pregnancy and implantation rates.

On the other hand the analysis of oocyte maturation might be of great importance in predicting successful fertilization and embryo development. As far as oocyte morphological criteria, several have been claimed to correlate with outcome, including polar body morphology [8]; cytoplasm appearance [9], and more recently zona pellucida thickness, appearance and birefringence [10]–[12] and the position or shape of the spindle [13]. Also in this case biochemical markers helping in identifying oocytes completing *in vitro* maturation would be very interesting in IVF approaches. Markers of oocyte maturation are the presence of activated mitochondria and the ability to mobilize and release calcium for internal stores [14].

In this paper we analyze the release by oocytes and in vitro fertilized human embryos of proteins involved in inflammation, including several cytokines, chemokines and soluble Intercellular Adhesion Molecule 1 (sICAM-1). This study was carried on using three independent methodologies, such as ELISA, Bio-Plex assay [15], [16], and Western blotting.

## Results

### Release of cytokines, chemokines and ICAM-1 by human embryos

We first performed a preliminary screening of 11 embryos using premixed multiplex beads of the Human 27-Plex Panel and the ICAM-1 Bio-Plex kit, obtaining the following results. IL-1β, IL-2, IL-4, IL-5, IL-10, IL-12 (P70), IL-15, IL-17, Basic FGF, G-CSF, GM-CSF, IFN-γ, MIP-1α, TNF-α were not present or undetectable in the analyzed supernatants. Presence of IL-1rα, IL-6, IL-7, IL-8, IL-9, IL-13, Eotaxin, IP-10, MCP-1 (MCAF), MIP-1β, PDGF-BB, RANTES, VEGF, ICAM-1 were detectable in 11, 1, 1, 10, 1, 1, 7, 1, 1, 1, 1, 1, 4, 11 embryos respectively. The only proteins present in the supernatant of all the screened embryos were ICAM-1 and IL-1rα. However, only ICAM-1 was expressed at high levels. In additional experiments on other IVS embryos (not included in this paper) we never found absence of ICAM-1 release, with the exception of few damaged embryos (data not shown).

### Quantization of sICAM-1: ELISA and Bio-Plex assay

**Figure 1:**
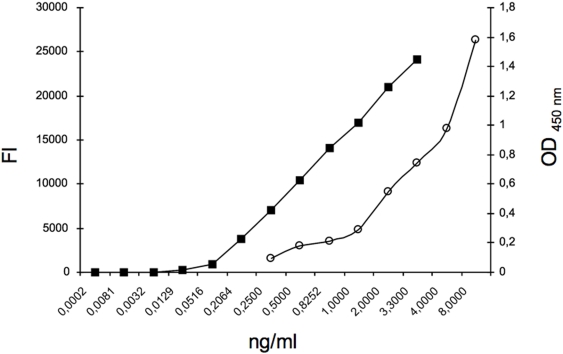
ELISA and Bio-Plex standard curves (white circles and black square respectively) have been obtained with 50 µl of ICAM-1 standard reagent at the concentrations of 0.25, 0.5, 1, 2, 4, 8 ng/ml or 0.0002, 0.00081, 0.0032, 0.0129, 0.0516, 0.20638, 0.82522, 3.3 ng/ml as indicated. FI: fluorescence intensity values. OD 450 nm: optic density at 450 nm wavelength.

In Figure 1 representative analysis is shown demonstrating that levels of ICAM-1 standards are detectable following both ELISA and the Bio-Plex assay. As expected, however, the Bio-Plex assay is more sensitive than ELISA. This is of course important for analysis of single cells, including oocytes. Accordingly, Bio-Plex analysis was chosen for studies involving human oocytes and fertilized embryos.

### Comparison of sICAM-1 production in mature and immature oocytes and in vitro fertilized embryos

**Figure 2:**
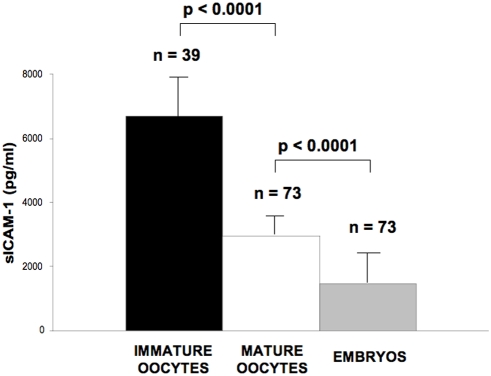
sICAM-1 release in immature oocytes (black box) compared to mature (white box) oocytes and to in vitro fertilized embryo (grey box). Oocytes were individually cultured in a 4-well culture dish as reported in the methods section. Following the maturation period 250 µl of supernatants were collected from each culture system and stored at −20°C until being tested for the presence released proteins. Mature and immature oocytes were identified, one by one, evaluating the presence or absence of the first polar body. In vitro fertilized embryos were individually cultured in 4-well culture dishes and 250 µl of supernatants collected from each embryo culture and stored at −20°C until being tested for the presence of released proteins. * Student t Test.

Figure 2 reports a sharp difference in sICAM-1 levels among immature and mature oocytes and fertilized embryos. The average sICAM-1 production by immature (n = 39) and mature (n = 73) oocytes was 6711.5±1502.4 and 2987±103.7 pg/ml/24 hours (mean±SD), respectively (Figure 2). This difference was very reproducible and statistically significant (Student t Test, p<0.0001). In addition, the levels of release of sICAM-1 levels by mature oocytes and in vitro fertilized embryos (n = 73), 1486.8±164.2 pg/ml/24 hours, were also found to be significantly different (Student t Test, p<0.0001) (Figure 2). Therefore, it appears that the release of sICAM-1 has a clear tendency to decrease from immature embryos, to mature embryos and to fertilized embryos.

**Figure 3:**
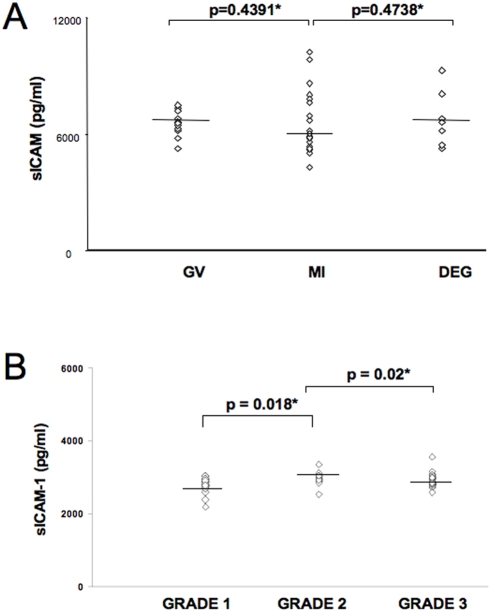
Western blotting analysis. The anti-ICAM-1 MoAb was used for the detection. a: standard positive control loaded at 8000 pg; b: plasma sample loaded at 10000 pg, accordingly to ELISA detection; c: medium negative control; d: mature oocyte supernatant loaded at 35 pg accordingly to ELISA detection; e: immature oocyte supernatant loaded at 100 pg accordingly to ELISA detection; M: protein ladder.

The presence of sICAM-1 molecules in oocytes culture supernatants was also analyzed by western blotting. The results obtained are shown in Figure 3. Standard positive ICAM-1 controls are shown in lanes “a” and “b”. As clearly evident, sICAM-1 is detectable both in mature (lane “d”) and immature (lane “e”) oocyte supernatants. In addition, sICAM-1 is present in mature oocytes in lower quantities in respect to immature oocytes, fully in agreement with the Bio-Plex data shown in Figure 3. These data were fully in agreement with ELISA assays (data not shown).

### Production of sICAM-1 in immature and mature oocytes

**Figure 4:**
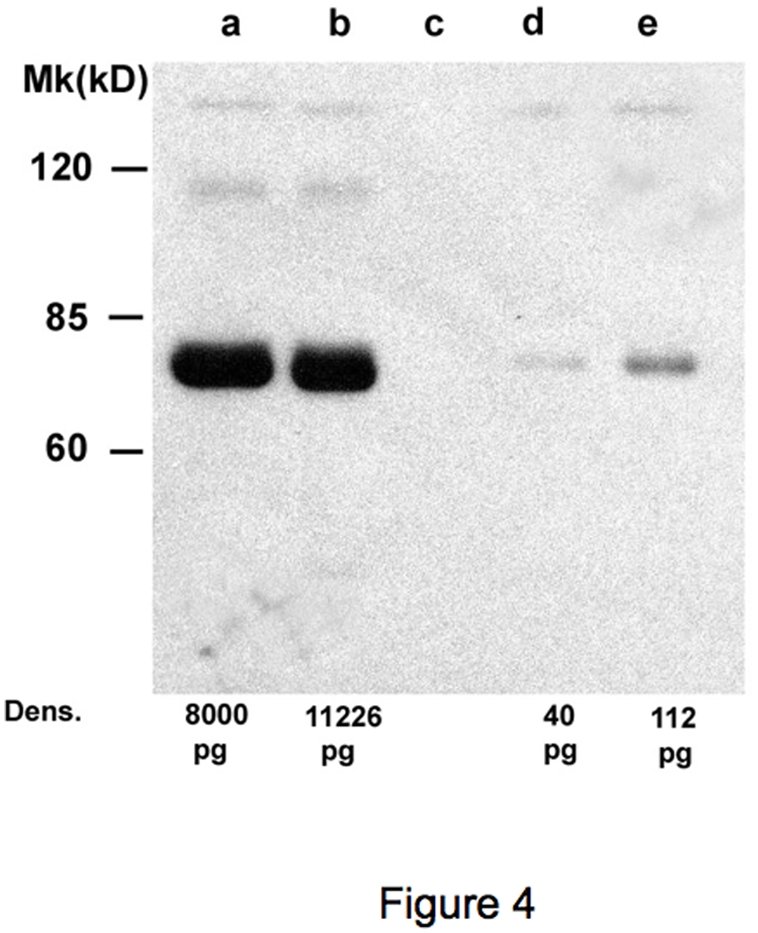
sICAM-1 levels in culture supernatants from immature (a) and mature oocytes (b). Immature oocytes were analysed individually for morphological characteristics to differentiate them as Metaphase I (MI), germinal vescicle (GV) and degenerated (DEG). (a). Mature oocytes were subdivided on the basis of the first polar body and cytoplasm characteristic in Grade 1, 2 and 3 (b). Preparation of oocyte supernatants was performed as described in the legend to Figure 2. * Student t Test.

Figure 4a reports the levels of sICAM-1 in immature oocytes at different maturation stages (MI, Metaphase I; GV, germinal vesicle; DEG, degenerated). The average released sICAM-1 was 5900 pg/ml/24 hours for MI oocytes, 6600 pg/ml/24 hours for GV oocytes and 6600 pg/ml/24 hour for DEG oocytes. The difference between sICAM-1 production by MI, GV and DEG immature oocytes was not statistically significant (Student t Test, p = NS) (Figure 3a).

Moreover, Figure 4b reports the analysis of ICAM-1 release in mature oocytes subdivided in grade 1, 2 and 3, as reported in the material and methods section. The average released sICAM-1 was 2804 pg/ml/24 hours for grade 1 oocytes, 2978 pg/ml/24 hours for grade 2 oocytes and 2923 pg/ml/24 hours for grade 1 oocytes (Figure 4b). Statistical analysis showed significant lower levels of sICAM-1 in grade 1 oocyte supernatants in comparison to grade 2 (Student t Test, p = 0.018) and grade 3 (p = 0.02) oocyte supernatants. Therefore, lower slCAM-1 levels in mature oocyte are predictive for the best grade oocytes (Grade 1).

### sICAM-1 levels and Embryo Grade

**Figure 5:**
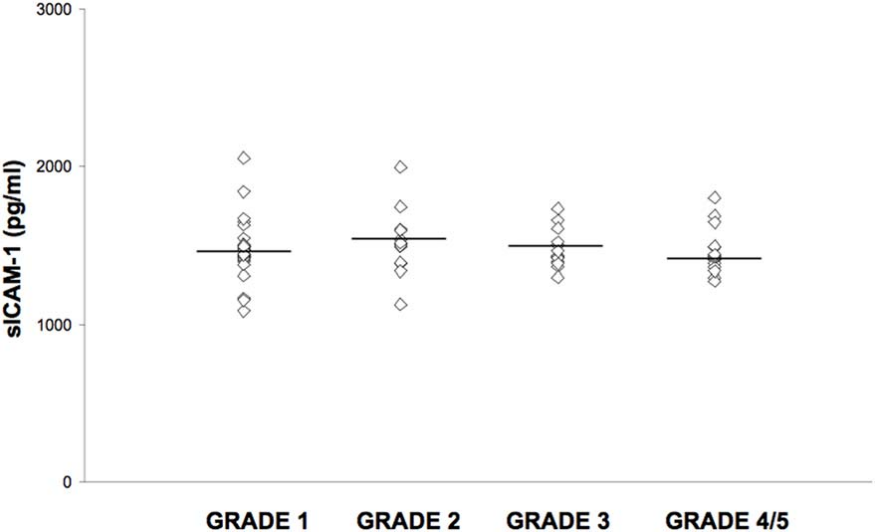
sICAM-1 levels in embryo culture supernatants subdivided into grades as reported in the Methods section.

Figure 5 represents sICAM-1 levels in embryo culture supernatants graded as reported in the Methods section. The average levels of sICAM-1 were 1476.3±187 pg/ml/24 hours in the 29 Grade 1 embryos; 1522.4±206 pg/ml/24 hours in the 13 of Grade 2; 1481±116 pg/ml/24 hours in the 15 of Grade 3; 1461.9±143.9 pg/ml/24 hours in the 16 Grade 4 and 5 embryos.

The differences between sICAM-1 production by Grade 1, 2, 3, 4 and 5 embryos were not statistically significant (Student t Test, p = NS).

### sICAM-1 levels in oocyte supernatants and pregnancy rate

Table 1 reports the sICAM-1 mean levels observed in mature oocyte and embryo culture supernatants subdivided for implantation and pregnancy outcome.

**Table 1:** sICAM-1 release, implantation outcome and pregnancy outcome.

	Implantation outcome		Pregnancy outcome	
	positive	negative	positive	negative
frequency (n)	7	35	4	38
sICAM-1 oocytes (pg/ml)	2865.4±107.7	2856.9±520	2858.4±117.6	2860.7±403.1
sICAM-1 embryos (pg/ml)	1424.1±142.5	1493.8±170.7	1463.5±127.5	1483.9±171.2

There were no statistical differences (Student t Test, p = NS) in sICAM-1 levels observed in the supernatant of oocytes and embryo with a negative or positive implantation and pregnancy rate (Table 1).

The relationship between oocyte grade (Figure 4b) and implantation/pregnancy rate was not investigated, since we were not able to associate the pregnancy event to a specific embryo. In fact, our IVF protocol, in order to achieve the highest probability of pregnancy and meet law restrictions [17], allows the transfer of three embryos that could originate from different grade oocytes.

### Comparison of sICAM-1 and sHLA-G levels in supernatants of oocytes using Bio-plex technology

**Figure 6:**
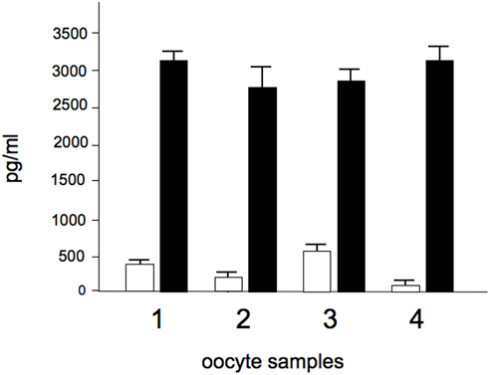
Comparison of levels of sICAM-1 (black boxes) and sHLA-G (white boxes) in supernatants of representative oocytes. For sHLA-G detection, covalent coupling of the anti-sHLAG antibodies to the carboxylated polystyrene microspheres (Bio-Rad, Hercules, CA, USA) was performed using the Bio-Plex amine coupling kit (Bio-Rad, Hercules, CA, USA). Bio-Plex assay was performed as elsewhere reported [2].

Since the release of soluble HLA-G (sHLA-G) molecules by in vitro fertilized embryos seems to help the morphological characterization in the selection of the most promising embryo obtained by IVF and has been proposed as a possible candidate marker for oocyte maturation [17] we compared the release of these two proteins in our samples. Representative analyses are shown in Figure 6, which clearly indicate that in human oocytes the release of sICAM-1 is far more efficient that release of sHLA-G molecules. In general, the release of sHLA-G molecules is very low in most of the oocytes employed. On the contrary, confident results are obtained studying sICAM-1, due to the high release of this protein by human oocytes.

## Discussion

The reduction of the number of fertilized oocytes and transferred embryos represents the main target of assisted reproductive medicine. During the last years, several studies have confirmed the significant relationship between the production of sHLA-G molecules by 48–72 hours early embryos and an increased implantation rate in IVF protocols [18]. As consequence, the detection of HLA-G modulation was suggested as a marker to identify the best embryos to be transferred. On the opposite, no suitable markers are available for the oocyte selection. The major finding of this paper is the detection, by a reliable technique, of soluble ICAM-1 molecules in the culture supernatants of human in vitro maturated oocytes and in vitro fertilized embryos. The data obtained showed a significant difference in sICAM-1 levels between immature and mature oocytes with significant higher amounts of sICAM-1 in the oocytes that failed to maturate. When the immature oocytes were morphologically classified in metaphase I, germinal vesicle and degenerated phenotypes we observed similar levels of sICAM-1 in the three groups. On the contrary, the mature oocytes subdivided into grade 1, 2 and 3 presented lower s-ICAM-1 levels in grade 1 group. Therefore, these results propose sICAM-1 levels as predictive for oocyte maturation and quality. Biochemical markers of the oocyte maturation are very important, due to the fact that (a) during in vitro oocyte maturation no ore than 50% of the oocytes isolated from a single woman reach grade 1 (Table 2); (b) only these oocytes are routinely considered for IVF. In addition, we like to point out that in some countries no embryo selection is allowed, only a limited number of oocytes are fertilized and all of the obtained embryos must be implanted [19].

**Table 2:** Details of the in vitro maturation procedure.

	Women (n = 42)
Age (years) (mean±SD)	35±3
Number of recovered oocytes per woman (mean±SD)	7±1
Mature oocytes per woman (%)	20–50

The culture supernatants of early embryos showed sICAM-1 levels lower in comparison to both mature and immature oocytes. Interestingly no significant differences were observed in sICAM-1 concentrations in the culture supernatants of early embryos subdivided into grades. These results underline the importance of sICAM-1 as a marker of the oocyte maturation process but not of the early embryos development.

This is the first report showing release of sICAM-1 in human oocytes and IVF human embryo. However the expression of ICAM-1 in human embryos is not surprising, when considering the implantation phase. In this context, ICAM-1 has been already presented as a protein involved in inflammation. In fact ICAM-1 knock-out mice do not develop inflammation and have less inflammatory cell infiltration [20], [21]. Mutations of ICAM-1 are associated with different diseases as infarct, biliary atresia, multiple sclerosis, obesity [21]–[24]. When the sICAM-1 levels are compared to sHLA-G, a soluble molecule involved in embryo implantation [3], sICAM-1 showed higher levels in oocyte supernatants than sHLA-G. These two molecules are both secreted by human oocytes but with a more efficient release of sICAM-1 than of sHLA-G molecules. In general, the release of sHLA-G molecules is very low in most of the oocytes employed. On the contrary, confident results are obtained studying sICAM-1, due to the high release of this protein by human oocytes.

The results obtained are confirmed using three independent methodologies, such as ELISA, Bio-Plex assay and Western blotting. Therefore, we propose this biochemical marker to be tightly linked to oocyte maturation. This finding is novel and, in our opinion, very important in the field of the selection of oocytes to be fertilized.

As known, the oocytes obtained under ovarian stimulation present a variable competence and although molecular approaches have been proposed [25], [26], the selection is still performed on morphological characteristics such as ploidy and chromosome/chromatin status. Since maturation of oocytes is so important for in vitro fertilization approaches, we suggest sICAM-1 to be a marker for testing different culture mediums under development by several laboratories to the aim to obtain optimal in vitro oocyte maturation.

In conclusion, our data encourage further studies from different laboratories/networks using ICAM-1 as a marker for a positive oocyte maturation.

## Materials and Methods

### Patients

The oocytes employed in this study were obtained from regularly cycling patients attending the Biogenesi Reproductive Medicine Centre of Monza, Italy, for an Assisted Reproduction Technique with In Vitro Maturation Protocol (IVM). Couples included in the trial had an indication to IVF procedure because of infertility due to male factor, tubal factor, stage I/II endometriosis, polycystic ovarian syndrome (PCO) or unexplained cause. All the women included had regular cycles of 26–35 days. A written informed consensus was obtained from all participating couples. We considered just one cycle per couple, and after maturation process we used from one to three oocytes according to the Italian Law 40 on IVF. Following these criteria, 42 women were recruited for the study. Women characteristics are reported in Table 2.

Oocyte recovery was performed by means of transvaginal ultrasonound–guided follicle aspiration, using a single lumen aspiration needle (Gynetics cod. 4551-E2 Ø17- gauce 35 cm) connected to a vacuum pump (Craft Pump pressure 80–90 mmHg). The retrieved oocytes were surrounded by granulosa cells forming a structure known as the cumulus ophorus complex (COC). The COCs were washed with prewarmed Flushing Medium with heparin (Medi-Cult product n. 10760125, Denmark).

The COCs, that for easiness we will define oocytes, were detected under a stereomicroscope, examined and classified on the basis of their morphology. Oocytes with signs of mechanical damage or atresia were discarded.

Immature oocytes were individually cultured in a 4-well culture dish with 0,5 ml of IVM Medium (vial 2 of IVM system medium; Medicult no. 82214010, Denmark) supplemented with rec–FSH 0,075 IU/ml (Serono, Italy), hCG 0,1 IU/ml (Serono, Italy) and 10% Serum Protein Substitute (SPS no. 3010– Sage Media- USA) for other 30 hrs.

Following the maturation period, 250 µl of supernatants were collected from each culture system containing a single oocyte and stored at −20°C until being tested for the presence released proteins.

The oocytes were then classified, one by one, evaluating the presence of the first polar body to confirm Metaphase II stage and their morphological characteristics.

Immature oocytes were classified as Metaphase I (MI), germinal vesicle (GV) and degenerated (DEG) whereas mature oocytes were classified on the first polar body and cytoplasm characteristics in Grade1: homogenous cytoplasm and round polar body; Grade 2: oocyte with variations in color or cytoplasm granularity and/or presence of inclusions, vacuoles or retractable bodies, but a round polar body: Grade 3: oocyte with variation in color or cytoplasm granularity and/or presence of inclusions, vacuoles or retractable bodies with a fragmented polar body.

Embryos were graded accordingly to cleavage (cell number) and cytoplasmic fragmentation. Embryos were graded as follows on Day 3: Grade 1, blastomeres have equal size and no cytoplasmic fragmentation; Grade 2, blastomeres have equal size and minor cytoplasmic fragmentation involving <10% of the embryo; Grade 3, blastomeres have unequal size and fragmentation involving 10–20% of the embryo; Grade 4, blastomeres have equal or unequal size, and moderate to significant cytoplasmic fragmentation covering 20–50% of the embryo; and Grade 5, few blastomeres and severe fragmentation covering ≥50% of the embryo [17].

### Measurement of sICAM-1 levels by enzyme-immunosorbent assay (ELISA)

sICAM-1 concentrations were analyzed in triplicate on 1∶2 diluted oocyte culture supernatants by the commercially available sICAM-1 kit (Diaclone, Besancon, FR) with a detection limit of 0.25 ng/ml.

### Western blotting

The presence of sICAM-1 molecules in oocyte culture supernatants was analyzed by Western Blot. Briefly, concentrated and albumin depleted (Enchant Life Science kit, Pall Corporation, MI, US) oocyte culture supernatants were loaded on 8% SDS-polyacrylamide gel, electrophoresed at 80 V for 2 hours and blotted onto PVDF membrane (Immobilon-P Millipore, Billerica, MA, US) by electrotransfer at 100 V for 45 minutes in 25 mM Tris Buffer, 190 mM Glycine, 2% SDS and 20% (V/V) Methanol. Blocking was carried out with 5% nonfat dry milk, Tris 100 mM pH 7.5, NaCl 150 mM over night at 4°C. After two washes, the membrane was incubated with monoclonal mouse-anti-human ICAM-1 (10 µg/ml) (Genzyme, MA, USA) for 3 hours at room temperature with gentle shaking. The sICAM-1 molecules were detected using Protein-G HRP (BioRad, Hercules, CA, US) at dilution of 1∶5000 in 10 mM Tris pH 8.0, 150 mM NaCl, 0.1% Tween 20. Reactions were developed by chemiluminescence with SuperSignal enhanced chemiluminescence kit (Super Signal West Pico system, Pierce, Rockford, IL, US) and captured by Chemiluminescence Imaging Geliance 600 (PerkinElmer, CT, USA). The ELISA standard (sICAM-1 kit (Diaclone, Besancon, FR)) and a plasma sample were used as positive control, the culture medium alone as negative control. The molecular weights were determined with the BenchMark (Invitrogen, CA, US) pre-stained protein ladder (range 10–200 kD). Densitometric analysis was performed with the Gene Tools software (PerkinElmer, CT, USA).

### Cyto/chemokines and ICAM-1 profiles

Cytokines and chemokines presence were measured in embryo culture supernatants by Bio-Plex cytokine assay (Bio-Rad Laboratories, Hercules, CA) [15], [16] described by the manufacturer. The Bio-Plex cytokine assay is designed for the multiplexed quantitative measurement of multiple cytokines in a single well using as little as 50 µl of sample. In our experiments, we used the premixed multiplex beads of the and Bio-Plex Human Cytokine singleplex Assay ICAM-1 (Bio-Rad, Cat. no. XF0-000003N) and Bio-Plex human cytokine Human 27-Plex Panel (Bio-Rad, Cat. no. 171-A11127) which included twenty-seven cytokines [IL-1β, IL-1rα, IL-2, IL-4, IL-5, IL-6, IL-7, IL-8, IL-9, IL-10, IL-12 (P70), IL-13, IL-15, IL-17, Basic FGF, Eotaxin, G-CSF, GM-CSF, IFN-γ, IP-10, MCP-1 (MCAF), MIP-1α, MIP-1β, PDGF-BB, RANTES, TNF-α, VEGF]. Briefly, 50 µl of cytokine/chemokine and ICAM-1 standards or samples (supernatants from IVF human embryos) were incubated with 50 µl of anti-cytokine/chemikine/ICAM-1 conjugated beads in 96-well filter plates for 30 min at room temperature with shaking. Plates were then washed by vacuum filtration three times with 100 µl of Bio-Plex wash buffer, 25 µl of diluted detection antibody were added, and plates were incubated for 30 min at room temperature with shaking. After three filter washes, 50 µl of streptavidin-phycoerythrin was added, and the plates were incubated for 10 min at room temperature with shaking. Finally, plates were washed by vacuum filtration three times, beads were suspended in Bio-Plex assay buffer, and samples were analyzed on a Bio-Rad 96-well plate reader using the Bio-Plex Suspension Array System and Bio-Plex Manager software (Bio-Rad Laboratories, Hercules, CA).

### Statistical analysis

Statistical analysis was conducted using the Stat View software package (SAS Institute Inc, Cary, NC, US). The data were analyzed by the Student t test for unpaired samples. Statistical significance was assumed for p<0.05 (two tailed).

## References

[1] Deonandan R, Campbell MK, Østbye T, Tummon I (2000). Toward a more meaningful in vitro fertilization success rate.. J Assist Reprod Genet.

[2] Rebmann V, Switala M, Eue I, Schwahn E, Merzenich M, Grosse-Wilde H (2007). Rapid evaluation of soluble HLA-G levels in supernatants of in vitro fertilized embryos.. Human Immunol.

[3] Fuzzi B, Rizzo R, Criscuoli L, Noci I, Melchiorri L (2002). HLA-G expression in early embryos is a fundamental prerequisite for the obtainment of pregnancy.. European J Immunol.

[4] Noci I, Fuzzi B, Rizzo R, Melchiorri L, Criscuoli L (2005). Embryonic soluble HLA-G as a marker of developmental potential in embryos.. Human Reprod.

[5] Sher G, Keskintepe L, Batzofin J, Fisch J, Acacio B (2005). Influence of early ICSI-derived embryo sHLA-G expression on pregnancy and implantation rates: a prospective study.. Human Reprod.

[6] Desai N, Filipovits J, Goldfarb J (2006). Secretion of soluble HLA-G by day 3 human embryos associated with higher pregnancy and implantation rates: assay of culture media using a new ELISA kit.. Reprod Biomed Online.

[7] Yie SM, Balakier H, Motamedi G, Librach CL (2005). Secretion of human leukocyte antigen-G by human embryos is associated with a higher in vitro fertilization pregnancy rate.. Fertil Steril.

[8] Ebner T, Yaman C, Moser M, Sommergruber M, Feichtinger O, Tews G (2000). Prognostic value of first polar body morphology on fertilization rate and embryo quality in intracytoplasmic sperm injection.. Human Reproduction.

[9] Ebner T, Moser G, Tews G (2006). Is oocyte morphology prognostic of embryo developmental potential after ICSI?. Reproductive BioMedicine Online.

[10] Shen Y, Stalf T, Mehnert C, Eichenlaub-Ritter U, Tinneberg HR (2005). High magnitude of light retardation by the zona pellucida is associated with conception cycles.. Human Reproduction.

[11] Montag M, Schimming T, Köster M, Zhou C, Dorn C (2008). Oocyte zona birefringence intensity is associated with embryonic implantation potential in ICSI cycles.. Reproductive BioMedicine Online.

[12] Pascale M-P, Chretien M-F, Malthiery Y, Reynier P (2007). Mitochondrial DNA in the oocyte and the developing embryo.. Current Topics in Developmental Biology.

[13] Madaschi C, de Souza Bonetti TC, de Almeida Ferreira Braga DP, Pasqualotto FF, Iaconelli A, Borges E (2008). Spindle imaging: a marker for embryo development and implantation.. Fertil Steril.

[14] Dumollard R, Duchen M, Carroll J (2007). The role of mitochondrial function in the oocyte and embryo.. Curr Top Dev Biol.

[15] de Jager W, te Velthuis H, Prakken BJ, Kuis W, Rijkers GT (2003). Simultaneous detection of 15 human cytokines in a single sample of stimulated peripheral blood mononuclear cells.. Clin Diagn Lab Immunol.

[16] Kerr JR, Cunniffe VS, Kelleher P, Smith J, Vallely PJ, Will AM (2004). Circulating cytokines and chemokines in acute symptomatic parvovirus B19 infection: negative association between levels of pro-inflammatory cytokines and development of B19-associated arthritis.. J Med Virol.

[17] Veeck L (1999). An Atlas of Human Gametes and Conceptuses.

[18] Rizzo R, Fuzzi B, Stignani M, Criscuoli L, Melchiorri L (2007). Soluble HLA-G molecules in follicular fluid: a tool for oocyte selection in IVF?. J Reprod Immunol.

[19] Fineschi V, Neri M, Turillazzi E (2005). The new Italian law on assisted reproduction technology.. J Med Ethics.

[20] Hallahan DE, Virudachalam S (1997). Intercellular adhesion molecule 1 knockout abrogates radiation induced pulmonary inflammation.. Proc Natl Acad Sci USA.

[21] Wang HW, Babic AM, Mitchell HA, Liu K, Wagner DD (2005). Elevated soluble ICAM-1 levels induce immune deficiency and increase adiposity in mice.. FASEB J.

[22] Arikan C, Berdeli A, Kilic M, Tumgor G, Yagci RV, Aydogdu S (2008). Polymorphisms of the ICAM-1 gene are associated with biliary atresia.. Dig Dis Sci.

[23] van den Borne SW, Narula J, Voncken JW, Lijnen PM, Vervoort-Peters HT (2008). Defective intercellular adhesioncomplex in myocardium predisposes to infarctrupture in humans.. J Am Coll Cardiol.

[24] Mousavi SA, Nikseresht AR, Arandi N, Borhani Haghighi A, Ghaderi A (2007). Intercellular adhesion molecule-1 gene polymorphism in Iranian patients withmultiple sclerosis.. Eur J Neurol.

[25] Coticchio G, Sereni E, Serrao L, Mazzone S, Iadarola I, Borini A (2004). What criteria for the definition of oocyte quality?. Annals New York Academy of Science.

[26] Patrizio P, Fragouli E, Bianchi V, Borini A, Wells D (2007). Molecular methods for selection of the ideal oocyte.. Reproductive BioMedicine Online.

